# Taxonomic notes on *Stemmops* O. Pickard-Cambridge, 1894 (Araneae, Theridiidae) of China

**DOI:** 10.3897/BDJ.11.e106208

**Published:** 2023-06-26

**Authors:** Fengyuan Li, Yejie Lin, Fan Gao, Yanbin Yao, Shuqiang Li

**Affiliations:** 1 State Key Laboratory of Protein and Plant Gene Research, School of Life Sciences, Peking University, Beijing 100871, China State Key Laboratory of Protein and Plant Gene Research, School of Life Sciences, Peking University Beijing 100871 China; 2 Hebei Key Laboratory of Animal Diversity, College of Life Science, Langfang Normal University, Langfang 065000, China Hebei Key Laboratory of Animal Diversity, College of Life Science, Langfang Normal University Langfang 065000 China; 3 Nanjing University, Nanjing 210023, China Nanjing University Nanjing 210023 China; 4 Jinshan College of Fujian Agriculture and Forestry University, Fuzhou 350002, China Jinshan College of Fujian Agriculture and Forestry University Fuzhou 350002 China; 5 Institute of Zoology, Chinese Academy of sciences, Beijing 100101, China Institute of Zoology, Chinese Academy of sciences Beijing 100101 China

**Keywords:** diagnosis, new species, spider, type

## Abstract

**Background:**

The theridiid spider genus *Stemmops* O. Pickard-Cambridge, 1894 includes 27 extant species and is distributed in America (23 spp.) and Asia (4 spp.). Three species, *S.forcipus* Zhu, 1998 (♂♀), *S.nigrabdomenus* Zhu, 1998 (♂) and *S.nipponicus* Yaginuma, 1969 (♂♀), are currently known from China.

**New information:**

Two new species of *Stemmops* are described from China: *S.atratus* sp. n. (♀, Jiangsu, Zhejiang) and *S.lini* sp. n. (♂♀, Fujian, Zhejiang). In addition, the previously unknown female of *S.nigrabdomenus* Zhu, 1998 is described. Photos and morphological descriptions are provided.

## Introduction

The comb-footed spider family Theridiidae Sundevall, 1833 comprises 124 genera and 2541 known extant species worldwide (World Spider Catalog 2023). *Stemmops* Gerstaecker, 1873 was described with *S.bicolor* O. Pickard-Cambridge, 1894 from Teapa, Tabasco, Mexico as its type species. This genus can be separated from other related genera by: 1) chelicerae lacking tooth, 2) large eyes relatively close to one another, 3) short, stout legs, 4) the presence of only two spermathecae and 5) the colulus replaced by two setae (Levi 1955).

*Stemmops* currently comprises 27 extant species, distributed in America (23 spp.) and Asia (4 spp.). Although the Chinese spider taxonomists have published a large number of papers during the 21^st^ century, due to the rich biodiversity of the country, many groups remain poorly studied (Li 2020, Li et al. 2021, Yao et al. 2021, Lu et al. 2022, Zhao et al. 2022). Regarding *Stemmops*, Chen and Zhang (1991) provided the first data on this genus from China, by reporting *S.nipponicus* Yaginuma, 1969 from Zhejiang. However, the figures in that article are extremely schematic and the specimens are lost; therefore, it is impossible to determine whether the species identification is correct. Zhu (1998) described two species and, since then, the study of *Stemmops* in China has remained at the stage of reporting new distribution data. Therefore, three *Stemmops* species are currently known from China: *S.forcipus* Zhu, 1998, *S.nigrabdomenus* Zhu, 1998 and *S.nipponicus* (Chen and Zhang 1991, Zhu 1998).

During the examination of Chinese *Stemmops* specimens, we found two new species, as well as the unknown female of *S.nigrabdomenus* from Guangxi, all of which are described and illustrated in this paper.

## Materials and methods

All specimens are preserved in 80% ethanol. The epigynes were cleared in trypsin enzyme solution to dissolve non-chitinous tissues. Specimens were examined under a Leica M205C stereomicroscope and photographed using an Olympus C7070 zoom digital camera (7.1 megapixels). Photos of the live specimens were taken with a Sony A7RIV digital camera equipped with a Sony FE 90 mm Goss lens. Photos were stacked with Helicon Focus® (Version 7.6.1) or Zerene Stacker® (Version 1.04) and processed in Adobe Photoshop CC2022®. The distribution map was generated with ArcGIS v.10.2 (ESRI Inc.).

All measurements are in millimetres (mm) and were obtained with an Olympus SZX16 stereomicroscope with a Zongyuan CCD industrial camera. All measurements of body lengths do not include the chelicerae. Eye sizes are measured as the maximum diameter from either the dorsal or frontal view. Leg measurements are given as follows: total length (femur, patella+tibia, metatarsus, tarsus). The terminology used in the text and figures follows Agnarsson (2004) with modifications.

The type material is deposited at the Institute of Zoology, Chinese Academy of Sciences in Beijing (**IZCAS**).

Abbreviations: **ALE** anterior lateral eye; **AME** anterior median eye; **C** conductor; **CD** copulatory duct; **CO** copulatory opening; **E** embolus; **EBA** embolic basal apophysis; **EBF** embolic basal flat extension; **EBP** embolic basal process; **ETA** embolic terminal apophysis; **FD** fertilisation duct; **H** hood; **MA** median apophysis; **Se** septum; **PLE** posterior lateral eye; **PME** posterior median eye; **S** spermatheca; **SD** sperm duct; **ST** subtegulum; **T** tegulum; **TTA** theridiid tegular apophysis.

## Taxon treatments

### Stemmops atratus

Lin & S. Li sp. nov.

542BE86F-32C6-5CF9-9BD2-935CF8006995

68A42A58-3FC8-4CEC-8783-47A890848A6E

#### Materials

- **Type status:** Holotype. **Occurrence:** catalogNumber: IZCAS-Ar44589; recordedBy: Fan Gao; individualCount: 1; sex: female; lifeStage: adult; occurrenceID: 6D0CB4BA-4B57-5C31-B87A-75A9CEE400FB; **Taxon:** scientificName: *Stemmopsatratus*; **Location:** country: China; stateProvince: Jiangsu; locality: Nanjing City, Xuanwu District, Zhongshan; verbatimElevation: 74 m; decimalLatitude: 32.078; decimalLongitude: 118.837; **Identification:** identifiedBy: Yejie Lin; dateIdentified: 2023; **Event:** year: 2019; month: 6; day: 18
- **Type status:** Paratype. **Occurrence:** catalogNumber: IZCAS-Ar44590; recordedBy: Fan Gao; individualCount: 1; sex: female; lifeStage: adult; occurrenceID: CF399303-716E-594B-B4B7-89A38315A649; **Taxon:** scientificName: *Stemmopsatratus*; **Location:** country: China; stateProvince: Jiangsu; locality: Nanjing City, Xuanwu District, Zhongshan; verbatimElevation: 74 m; decimalLatitude: 32.078; decimalLongitude: 118.837; **Identification:** identifiedBy: Yejie Lin; dateIdentified: 2023; **Event:** year: 2019; month: 6; day: 18

#### Description

Female. Total length 2.85; carapace 0.94 long, 0.77 wide, opisthosoma 1.93 long, 1.45 wide. Eye sizes and interdistances: AME 0.05, ALE 0.06, PME 0.06, PLE 0.05, AME–AME 0.02, AME–ALE 0, PME–PME 0.02, PME–PLE 0.02, AME–PME 0.05, ALE–PLE 0.01. Endites almost as long as wide. Leg measurements: I 3.69 (1.05, 1.27, 0.80, 0.57), II 2.77 (0.79, 0.91, 0.55, 0.52), III 2.59 (0.74, 0.78, 0.51, 0.56), IV 3.93 (1.09, 1.26, 0.89, 0.69).

Colouration (Fig. 1a, Fig. 10a). Carapace dark-brown, covered with long sparse brown setae, eyes with black rings. Endites and labium green-brown. Sternum green-brown. Legs orange-brown, covered with sparse setae, more distinct on metatarsi and tarsi. Opisthosoma with two large white spots anteriorly and posteriorly, three pairs of small white symmetrical spots along the mid-line and a pair of white spots laterally, slightly larger than the small white spot in the middle. Spinnerets black.

**Figure 2.**
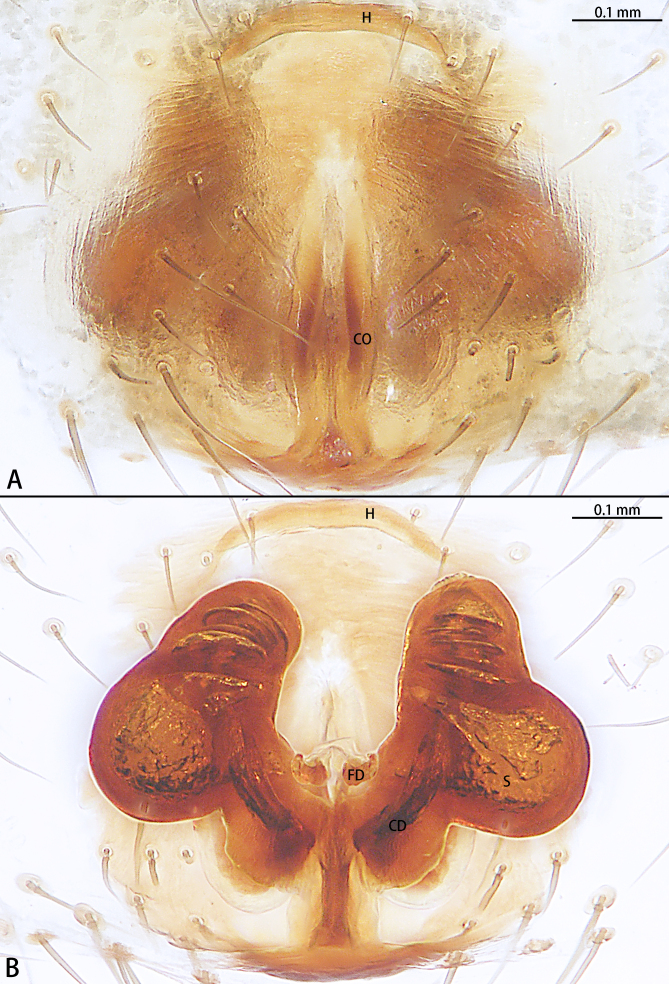
*Stemmopsatratus* sp. n., holotype female. **A** Epigyne, ventral; **B** Vulva, dorsal. Abbreviations: **CD** copulatory duct; **CO** copulatory opening; **FD** fertilisation duct; **H** hood; **S** spermatheca.

Epigyne (Fig. 2A and B). Epigynal plate almost as long as wide, with hood anteriorly, hood almost six times wider than long. Copulatory openings located on posterior portion of epigyne. First half of copulatory ducts slightly curved and second half coils around the middle of the spermathecae for four turns. Spermathecae kidney-shaped. Fertilisation ducts kidney-shaped.

Male. Unknown.

#### Diagnosis

The female of this new species is similar to that of *S.nipponicus* Yaginuma, 1969 by the split atrium (Fig. 2A). However, it can be distinguished by the presence of hood (Fig. 2A) (vs. absent) and the copulatory ducts coiled four times around the spermathecae (Fig. 2B) (vs. once in *S.nipponicus*). Furthermore, it differs by its habitus: *S.atratus* sp. n. lacks obscure yellow spots on the lateral view of opisthosoma (Fig. 1a, Fig. 10a Right) [vs. present in *S.nipponicus*, Yaginuma (1969) (Fig. 1d)].

#### Etymology

The specific epithet is derived from the Latin word *ātrātus*, meaning darkened, referring to the colour of the new species.

#### Distribution

China (Jiangsu, Zhejiang) (Fig. 11).

#### Biology

All specimens were collected under stones.

#### Notes

The dark venter of opisthosoma and epigynal hood indicate that the illustrated female of *S.nipponicus* in Jin (2018) is misidentified. Herein, we determine this species as *S.atratus* sp. n.

#### Compared species and material examined.

*Stemmopsnipponicus*, 10♂10♀, CHINA: *Beijing*: Chaoyang District, Olympic Forest Park, 40.0108°N, 101.2820°E, elevation 40 m, 7.VI.2022, Yejie Lin & Yunxiao Shen leg.

### Stemmops lini

Lin & S. Li sp. nov.

E461D6AC-9EB0-5B40-B898-9B4204314E7B

4CE8E6D6-7F68-4CA8-BC64-A75C5E7ED3F1

#### Materials

- **Type status:** Holotype. **Occurrence:** catalogNumber: IZCAS-Ar44591; recordedBy: Yanbin Yao; individualCount: 1; sex: male; lifeStage: adult; occurrenceID: 3D1902B0-5C90-5DB5-863B-8906B2D76E05; **Taxon:** scientificName: *Stemmopslini*; **Location:** country: China; stateProvince: Fujian; locality: Quanzhou City, Anxi County, Senshan Village, in Fujian Agriculture and Forestry University, near Gulujiao; verbatimElevation: 99 m; decimalLatitude: 25.0828; decimalLongitude: 118.2347; **Identification:** identifiedBy: Yejie Lin; dateIdentified: 2023; **Event:** year: 2023; month: 2; day: 26
- **Type status:** Paratype. **Occurrence:** catalogNumber: IZCAS-Ar44592; recordedBy: Yanbin Yao; individualCount: 1; sex: male; lifeStage: adult; occurrenceID: 411DF86F-3D59-5715-AB93-BA80039FA2CD; **Taxon:** scientificName: *Stemmopslini*; **Location:** country: China; stateProvince: Fujian; locality: Quanzhou City, Anxi County, Senshan Village, in Fujian Agriculture and Forestry University, near Gulujiao; verbatimElevation: 99 m; decimalLatitude: 25.0828; decimalLongitude: 118.2347; **Identification:** identifiedBy: Yejie Lin; dateIdentified: 2023; **Event:** year: 2023; month: 2; day: 26
- **Type status:** Paratype. **Occurrence:** catalogNumber: IZCAS-Ar44593; recordedBy: Yanbin Yao; individualCount: 1; sex: male; lifeStage: adult; occurrenceID: 411DF86F-3D59-5715-AB93-BA80039FA2CD; **Taxon:** scientificName: *Stemmopslini*; **Location:** country: China; stateProvince: Fujian; locality: Quanzhou City, Anxi County, Senshan Village, in Fujian Agriculture and Forestry University, near Gulujiao; verbatimElevation: 99 m; decimalLatitude: 25.0828; decimalLongitude: 118.2347; **Identification:** identifiedBy: Yejie Lin; dateIdentified: 2023; **Event:** year: 2023; month: 2; day: 26
- **Type status:** Paratype. **Occurrence:** catalogNumber: IZCAS-Ar44594; recordedBy: Yanbin Yao; individualCount: 1; sex: male; lifeStage: adult; occurrenceID: 411DF86F-3D59-5715-AB93-BA80039FA2CD; **Taxon:** scientificName: *Stemmopslini*; **Location:** country: China; stateProvince: Fujian; locality: Quanzhou City, Anxi County, Senshan Village, in Fujian Agriculture and Forestry University, near Gulujiao; verbatimElevation: 99 m; decimalLatitude: 25.0828; decimalLongitude: 118.2347; **Identification:** identifiedBy: Yejie Lin; dateIdentified: 2023; **Event:** year: 2023; month: 2; day: 26

#### Description

Male (holotype). Total length 1.71; carapace 0.78 long, 0.68 wide, opisthosoma 1.04 long, 0.57 wide. Eye sizes and interdistances: AME 0.04, ALE 0.04, PME 0.04, PLE 0.04, AME–AME 0.02, AME–ALE 0.01, PME–PME 0.02, PME–PLE 0.01, AME–PME 0.03, ALE–PLE 0. Endites almost as long as wide. Leg measurements: I 3.01 (0.82, 1.10, 0.63, 0.46), II 2.30 (0.61, 0.78, 0.44, 0.47), III 2.11 (0.53, 0.66, 0.43, 0.49), IV 3.33 (0.93, 1.13, 0.66, 0.61).

Colouration (Fig. 1b, Fig. 10b Left). Carapace yellow, edge black, eyes with black rings. Chelicerae yellow. Endites and labium yellow. Sternum paler yellow. Legs paler yellow, covered with sparse setae, more distinct on metatarsi and tarsi. Opisthosoma with two pale yellow spots anteriorly and posteriorly, two mountain-shaped pale-yellow spots in the middle, larger than the anterior and posterior yellow spots, pale yellow laterally. Spinnerets yellow.

**Figure 3.**
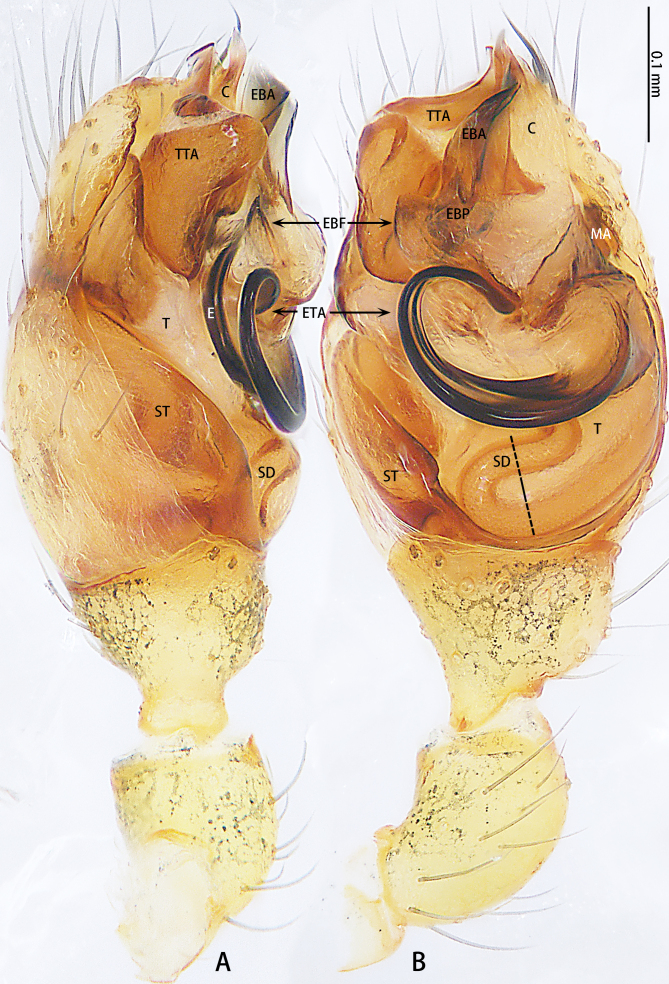
Left palp of *Stemmopslini* sp. n., holotype male. **A** Prolateral; **B** Ventral. Abbreviations: **C** conductor; **E** embolus; **EBA** embolic basal apophysis; **EBF** embolic basal flat extension; **EBP** embolic basal process; **ETA** embolic terminal apophysis; **MA** median apophysis; **SD** sperm duct; **ST** subtegulum; **T** tegulum; **TTA** theridiid tegular apophysis.

**Figure 4.**
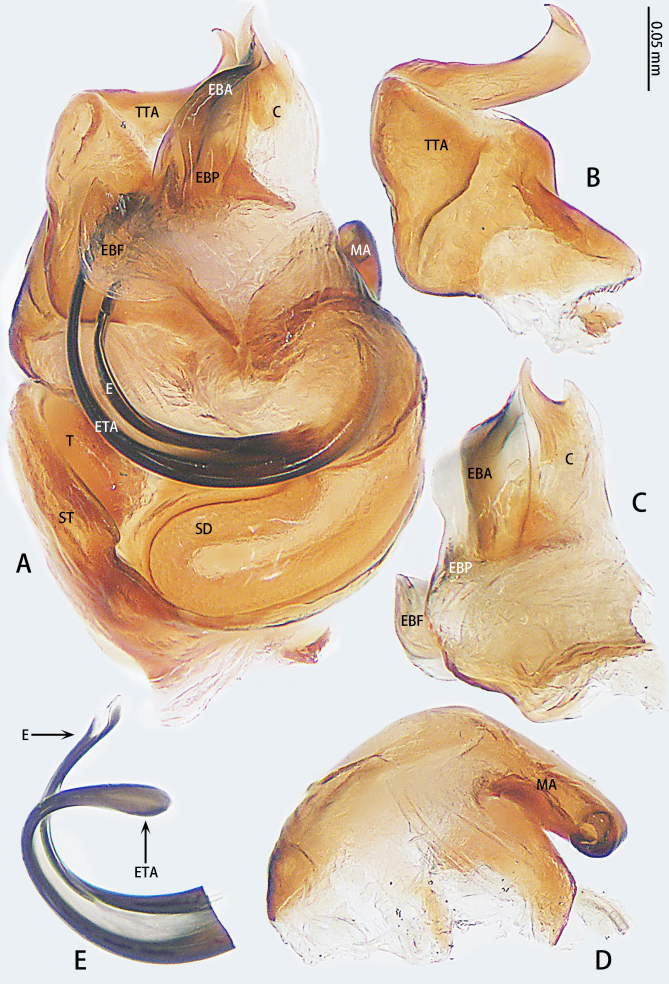
*Stemmopslini* sp. n., paratype male (**A**) and holotype male (**B**–**E**). **A** Bulb; **B** theridiid tegular apophysis; **C** Terminal apophysis, **D** Median apophysis; **E** Embolic tip. Abbreviations: **C** conductor; **E** embolus; **EBA** embolic basal apophysis; **EBF** embolic basal flat extension; **EBP** embolic basal process; **ETA** embolic terminal apophysis; **H**, hood; **MA** median apophysis; **SD** sperm duct; **ST** subtegulum; **T** tegulum; **TTA** theridiid tegular apophysis.

Palp (Fig. 3A, B, Fig. 4A–E). Patella almost as long as tibia. Cymbium almost as long as wide. Cymbium covered with sparse setae. Subtegulum on the lateral posterior side of the bulb, half encasing tegulum. Sperm duct S-shaped, the first bend obscured by embolus, not obvious, the second bend three times as wide as the diameter of the sperm duct. Median apophysis (Fig. 4D) hidden behind the conductor and tip with a hood. Theridiid tegular apophysis (Fig. 4B) divided into two parts by a translucent membrane: anterior part slightly curved, sickle-shaped, posterior almost quadrilateral. Conductor (Fig. 4C) tip pincer-shaped, located posterior to embolic basal apophysis. Embolic basal process (Fig. 4C) with two parts: embolic basal apophysis black with sharp end and folds, embolic basal flat extension semi-circular, flaky and transparent. Embolus (Fig. 4E) falciform, with spoon-shaped embolic terminal apophysis, connected by a membrane.

Female (IZCAS-Ar44593). Total length 2.14; carapace 0.85 long, 0.69 wide, opisthosoma 1.29 long, 0.75 wide. Eye sizes and interdistances: AME 0.03, ALE 0.05, PME 0.04, PLE 0.05, AME–AME 0.02, AME–ALE 0, PME–PME 0.02, PME–PLE 0.01, AME–PME 0.04, ALE–PLE 0.01. Endites almost as long as wide. Leg measurements: I 3.26 (0.93, 1.14, 0.65, 0.54), II 2.51 (0.72, 0.83, 0.45, 0.51), III 2.19 (0.58, 0.67, 0.42, 0.52), IV 3.18 (0.85, 1.05, 0.63, 0.65).

Colouration (Fig. 10b Right). Similar to that of male, except darker.

**Figure 5.**
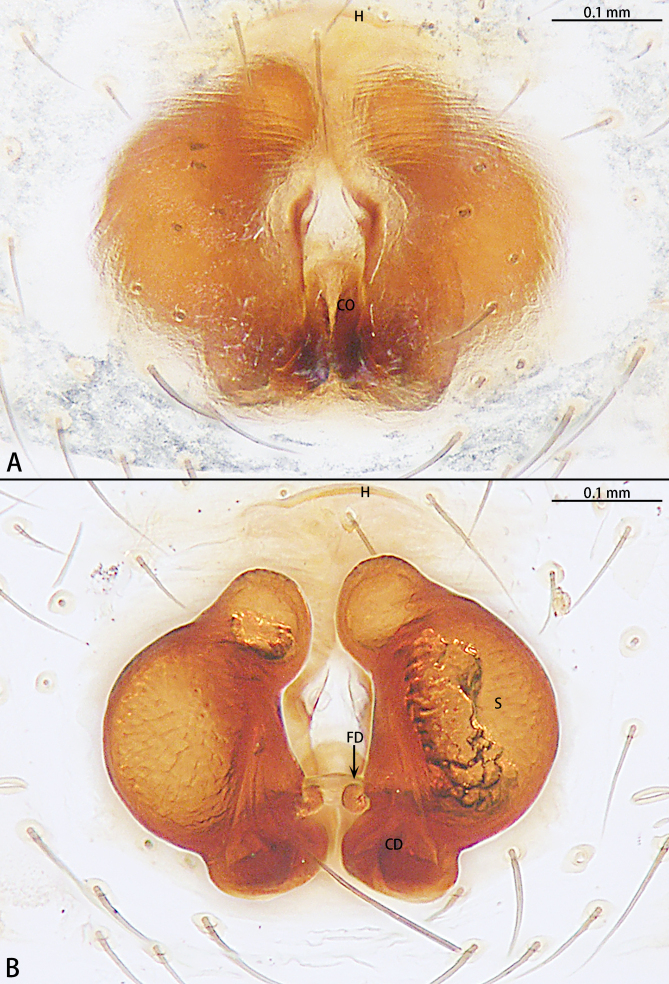
*Stemmopslini* sp. n., paratype female. **A** Epigyne, ventral; **B** Vulva, dorsal. Abbreviations: **CD** copulatory duct; **CO** copulatory opening; **FD** fertilisation duct; **H** hood; **S** spermatheca.

Epigyne (Fig. 5A, B). Epigynal plate almost as long as wide, with a weakly-developed hood anteriorly. Copulatory openings situated on posterior portion of epigyne. Copulatory ducts directly connected to spermathecae. Spermathecae kidney-shaped. Fertilisation ducts kidney-shaped.

#### Diagnosis

The male of this new species is similar to those of *S.forcipus* Zhu, 1998 and *S.nipponicus* by the median apophysis with depression (Fig. 4D), curved theridiid tegular apophysis (Fig. 3B, Fig. 4B, Fig. 6a, c), embolic basal apophysis strongly sclerotised and folded (Fig. 3B, Fig. 4C, Fig. 6a, c) and the presence of an embolic terminal apophysis (Fig. 4E, Fig. 7b, d, Fig. 8a, b, c). However, the new species can be distinguished from *S.forcipus* and *S.nipponicus* by the width of the sperm duct bend almost three times the diameter of the sperm duct (Fig. 3B) [vs. five times in *S.forcipus* and *S.nipponicus* (Fig. 6a, c)], presence of embolic basal apophysis and embolic basal flat extension (Fig. 3A, B, Fig. 4A, C) [vs. absent from embolic basal flat extension in *S.forcipus* and *S.nipponicus* (Fig. 6a, c)] and by the embolic terminal apophysis expanded and spoon-shaped (Fig. 4E) [vs. embolic terminal apophysis flat in *S.forcipus* and *S.nipponicus* (Fig. 7a, d)]. The female is similar to *S.nipponicus* by the split atrium (Fig. 5A). However, it can be distinguished from *S.nipponicus* by the length/width ratio of the atrium being almost 1:3 (Fig. 5A) (vs. 1:4 in *S.nipponicus*), the diameter of the spermathecae to the length of the copulatory ducts being almost 1:4 (vs. 1:2 in *S.nipponicus*) and by habitus, with *S.lini* sp. n. lacking white spots [vs. present in *S.nipponicus*, Yaginuma (1969) (*Fig. 1d*)], rather with round and mountain-shaped yellow spots (Fig. 1b).

#### Etymology

The species is named after Lin Zexu (1785–1850), a famous historical person born in Fujian who fought against imperialist aggression.

#### Distribution

China (Fujian, Zhejiang) (Fig. 11).

#### Biology

All specimens were collected under stones.

#### Notes

The presence of embolic basal apophysis and embolic basal flat extension and the spoon-shaped embolic terminal apophysis show the male of *S.nipponicus* in Jin (2018) is misidentified. Herein, we treat the male as *S.lini* sp. n.

#### Compared species and material examined.

*Stemmopsforcipus* Zhu, 1998, 5♂5♀, CHINA: *Yunnan*: Xishuangbanna, Mengla County, Lvshilin, 21.9033°N, 101.2820°E, elevation 608 m, 1–15.IV.2007, Guo Zheng leg.

### Stemmops nigrabdomenus

Zhu, 1998

D10A9535-01EC-5DFD-AFE3-11CAD72F0D18

4ZSMY

#### Materials

- **Type status:** Holotype. **Occurrence:** recordedBy: Yongqiang Zhang; individualCount: 1; sex: male; lifeStage: adult; occurrenceID: 7F54D174-ED02-5411-8E37-466B7FA9E31F; **Taxon:** scientificName: *Stemmopsnigrabdomenus*; **Location:** country: China; stateProvince: Guangxi; locality: Nanning City; **Identification:** identifiedBy: Mingsheng Zhu; dateIdentified: 1998; **Event:** year: 1991; month: 3; day: 15; eventRemarks: not examined; **Record Level:** institutionCode: MHBU
- **Type status:** Paratype. **Occurrence:** recordedBy: Yongqiang Zhang; individualCount: 2; sex: males; lifeStage: adult; occurrenceID: D23C8482-1AA3-5959-849E-C57121EDA32C; **Taxon:** scientificName: *Stemmopsnigrabdomenus*; **Location:** country: China; stateProvince: Guangxi; locality: Nanning City; **Identification:** identifiedBy: Mingsheng Zhu; dateIdentified: 1998; **Event:** year: 1991; month: 3; day: 15; eventRemarks: not examined; **Record Level:** institutionCode: MHBU
- **Type status:** Other material. **Occurrence:** recordedBy: Ming Yi; individualCount: 1; sex: male; lifeStage: adult; occurrenceID: 3938AA8D-43CB-52F3-BC8C-47BCE166FB8A; **Taxon:** scientificName: *Stemmopsnigrabdomenus*; **Location:** country: China; stateProvince: Guangxi; locality: Nanning City, Liangqing District, Nama Town; **Identification:** identifiedBy: Yejie Lin; dateIdentified: 2023; **Event:** year: 2019; month: 2; day: 15; **Record Level:** institutionCode: IZCAS
- **Type status:** Other material. **Occurrence:** catalogNumber: IZCAS-Ar44595; recordedBy: Chunxia Wang; individualCount: 1; sex: male; lifeStage: adult; occurrenceID: 51F7929A-C918-58A6-8251-4F319ADC53AA; **Taxon:** scientificName: *Stemmopsnigrabdomenus*; **Location:** country: China; stateProvince: Hainan; locality: Wenchang City, Tongguling Nature Reserve; verbatimElevation: 189 m; decimalLatitude: 19.6721; decimalLongitude: 111.0155; **Identification:** identifiedBy: Yejie Lin; dateIdentified: 2023; **Event:** year: 2007; month: 8; day: 17; **Record Level:** institutionCode: IZCAS
- **Type status:** Other material. **Occurrence:** catalogNumber: IZCAS-Ar44596; recordedBy: Qianle Lu; individualCount: 1; sex: female; lifeStage: adult; occurrenceID: 5DF6DC9E-C373-55E0-A44D-7EA3CF2EA477; **Taxon:** scientificName: *Stemmopsnigrabdomenus*; **Location:** country: China; stateProvince: Guangxi; locality: Yulin City, Beiliu City, Minle Town, Darong Mountain National Park; decimalLatitude: 22.8615; decimalLongitude: 110.2744; **Identification:** identifiedBy: Yejie Lin; dateIdentified: 2023; **Event:** year: 2022; month: 2; day: 15; **Record Level:** institutionCode: IZCAS

#### Description

Female (IZCAS-Ar44596). Total length 3.16; carapace 1.21 long, 1.04 wide, opisthosoma 2.08 long, 1.22 wide. Eye sizes and interdistances: AME 0.07, ALE 0.07, PME 0.07, PLE 0.07, AME–AME 0.06, AME–ALE 0.01, PME–PME 0.02, PME–PLE 0.01, AME–PME 0.06, ALE–PLE 0. Endites almost as long as wide. Leg measurements: I 5.13 (1.39, 1.78, 1.20, 0.76), II 3.67 (1.04, 1.14, 0.85, 0.64), III 3.47 (0.99, 1.09, 0.79, 0.60), IV 5.73 (1.59, 1.82, 1.46, 0.86).

Colouration (Fig. 1c, Fig. 10c Right). Carapace red-brown, covered with long sparse brown setae, black rings around PMEs. Chelicerae yellow. Endites and labium yellow. Sternum pale yellow. Legs red-brown, covered with sparse setae. Opisthosoma black, covered with sparse setae, with a white spot posteriorly. Spinnerets brown.

**Figure 9.**
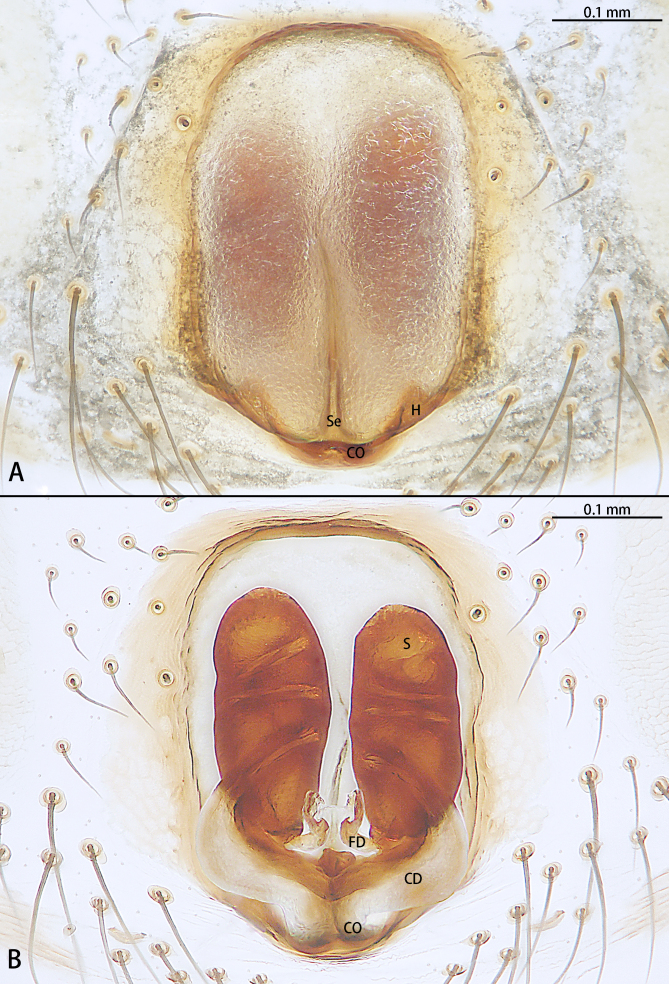
*Stemmopsnigrabdomenus*, female. **A** Epigyne, ventral; **B** Vulva, dorsal. Abbreviations: **CD** copulatory duct; **CO** copulatory opening; **FD** fertilisation duct; **H** hood; **Se** septum; **S** spermatheca.

Epigyne (Fig. 9A, B). Epigynal plate longer than wide, almost oval, with a pair of triangular hoods posteriorly. Septum emerges from a central position to the posterior. Copulatory openings located on posterior portion of epigyne. Copulatory ducts wide basally, curved and then coils around spermathecae for three turns. Spermathecae oval. Fertilisation ducts pincer-shaped.

Male. See Zhu (1998) (Figs 6b, 7c, 10c Left).

#### Diagnosis

The male can be easily recognised by the embolic basal apophysis white and transparent, without obvious sclerotisation (Fig. 6b) and the terminal of embolus not modified (Fig. 7c). Females can be distinguished by the large, oblong epigynal plate, with a pair of hoods posteriorly (Fig. 9A).

#### Distribution

**Figure 11.**
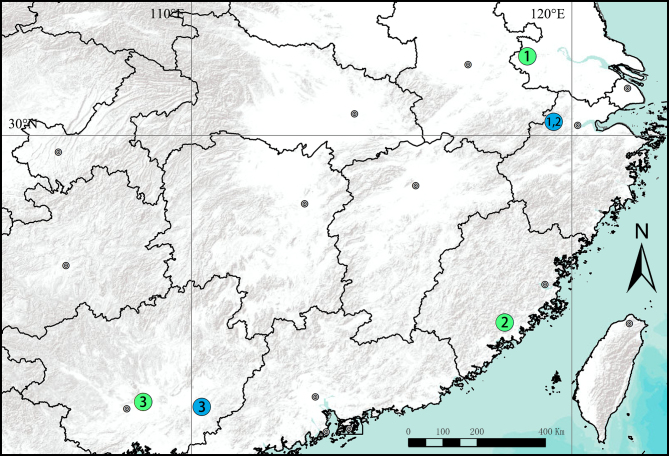
Distribution records of *Stemmops* species in China: **1** *S.atratus* sp. n.; **2** *S.lini* sp. n.; **3** *S.nigrabdomenus*. The green circles indicate type localities.

China (Guangxi, Hainan) (Fig. 11).

#### Notes

The female is described here for the first time.

## Supplementary Material

XML Treatment for Stemmops atratus

XML Treatment for Stemmops lini

XML Treatment for Stemmops nigrabdomenus

## Figures and Tables

**Figure 1a.**
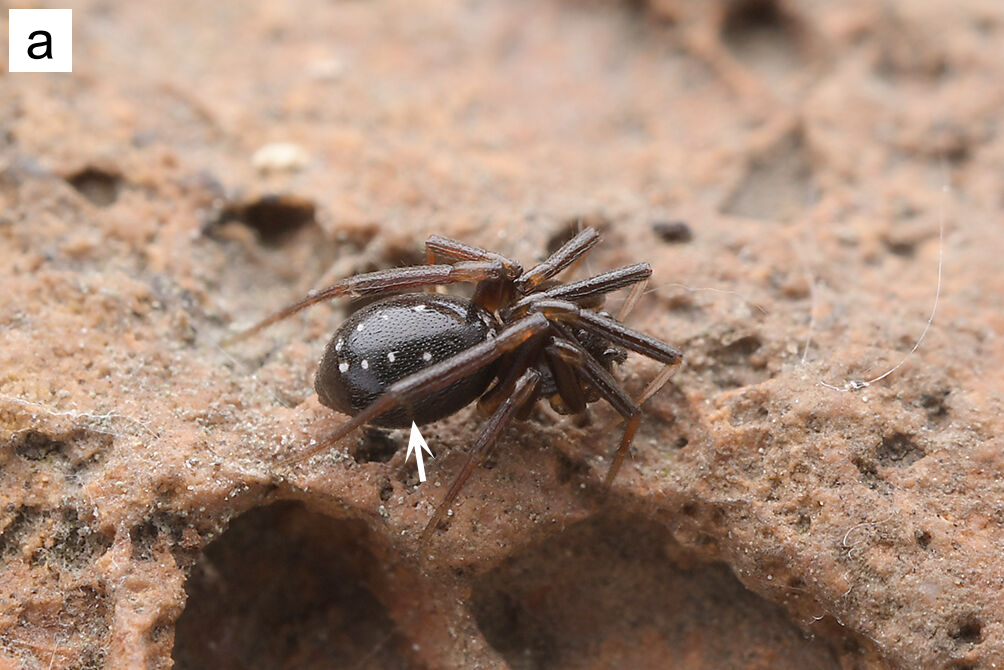
*S.atratus* sp. n., female holotype;

**Figure 1b.**
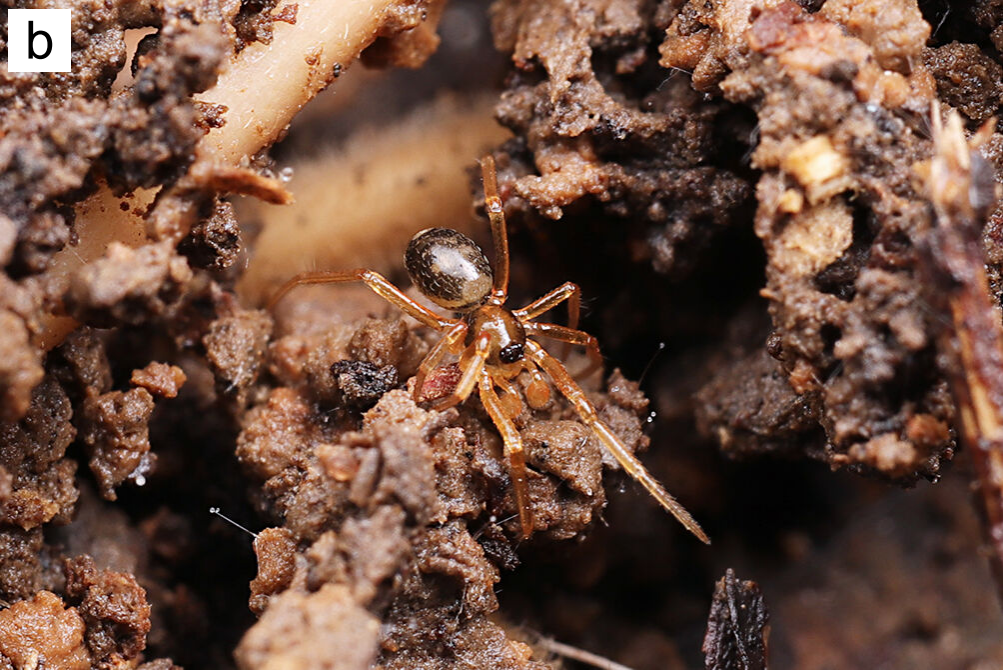
*S.lini* sp. n. subadult, male;

**Figure 1c.**
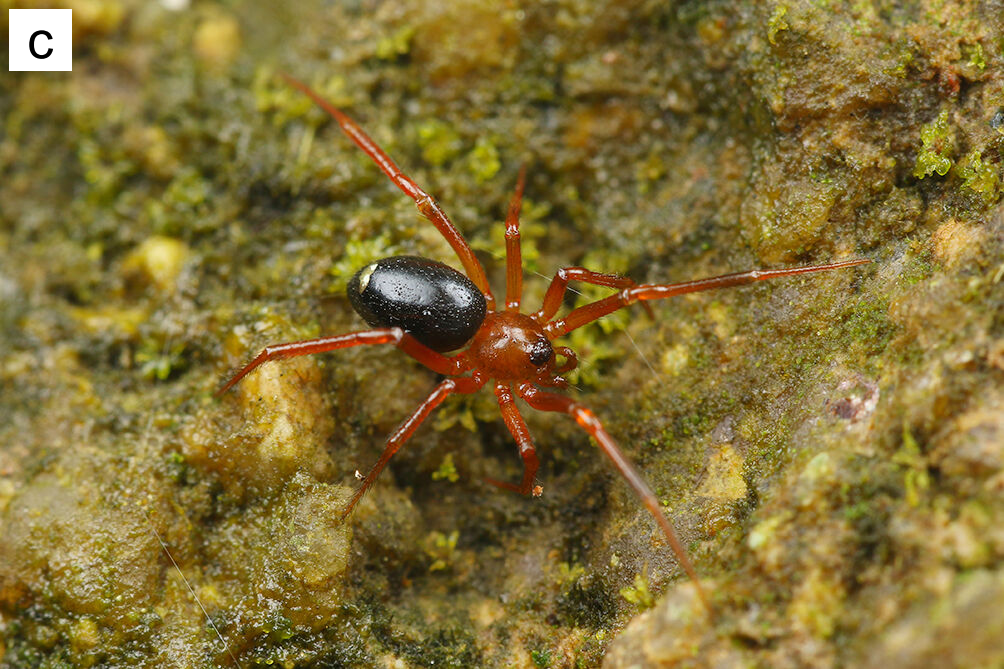
*S.nigrabdomenus*, female;

**Figure 1d.**
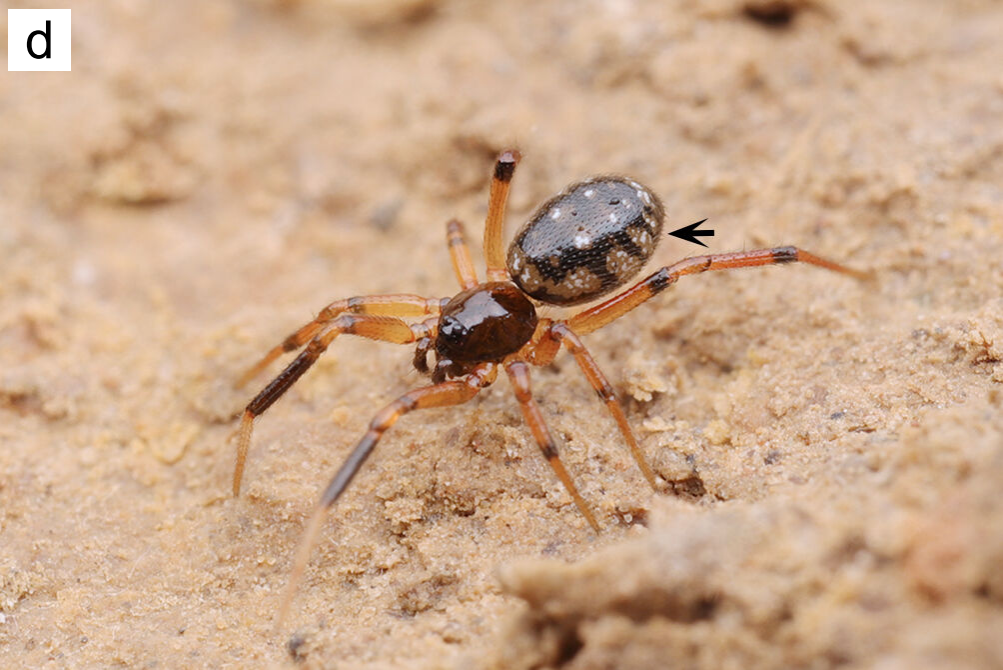
*S.nipponicus*, female.

**Figure 6a.**
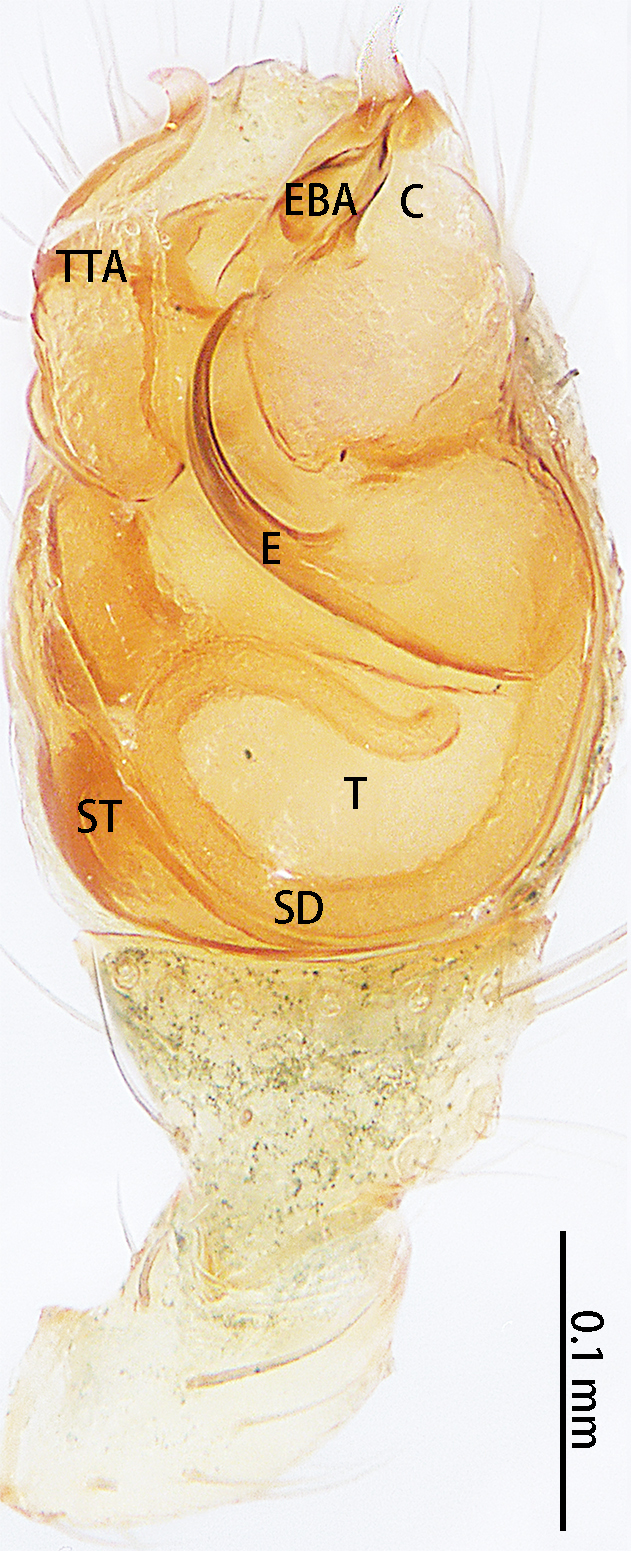
*S.forcipus*;

**Figure 6b.**
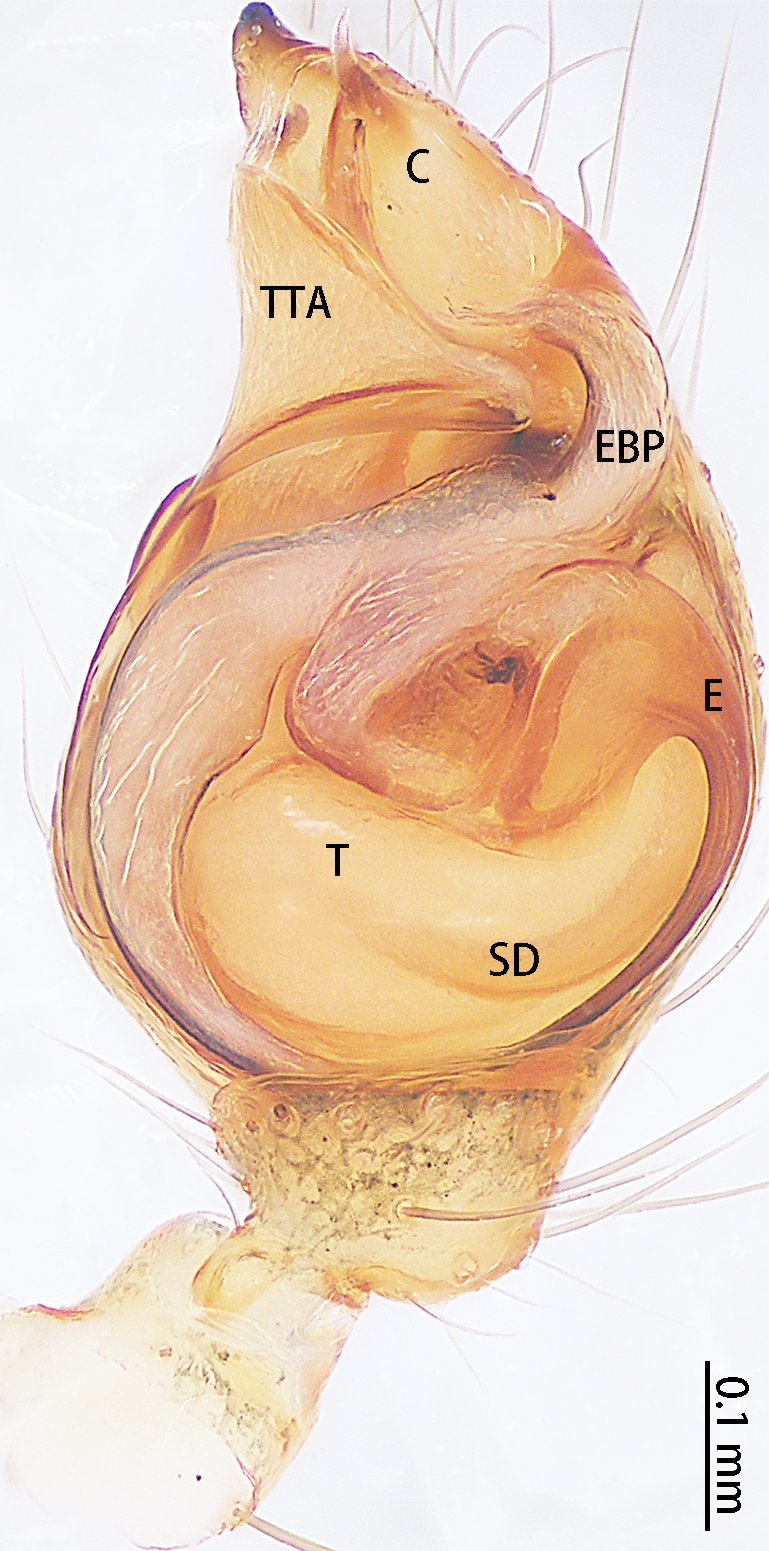
*S.nigrabdomenus*;

**Figure 6c.**
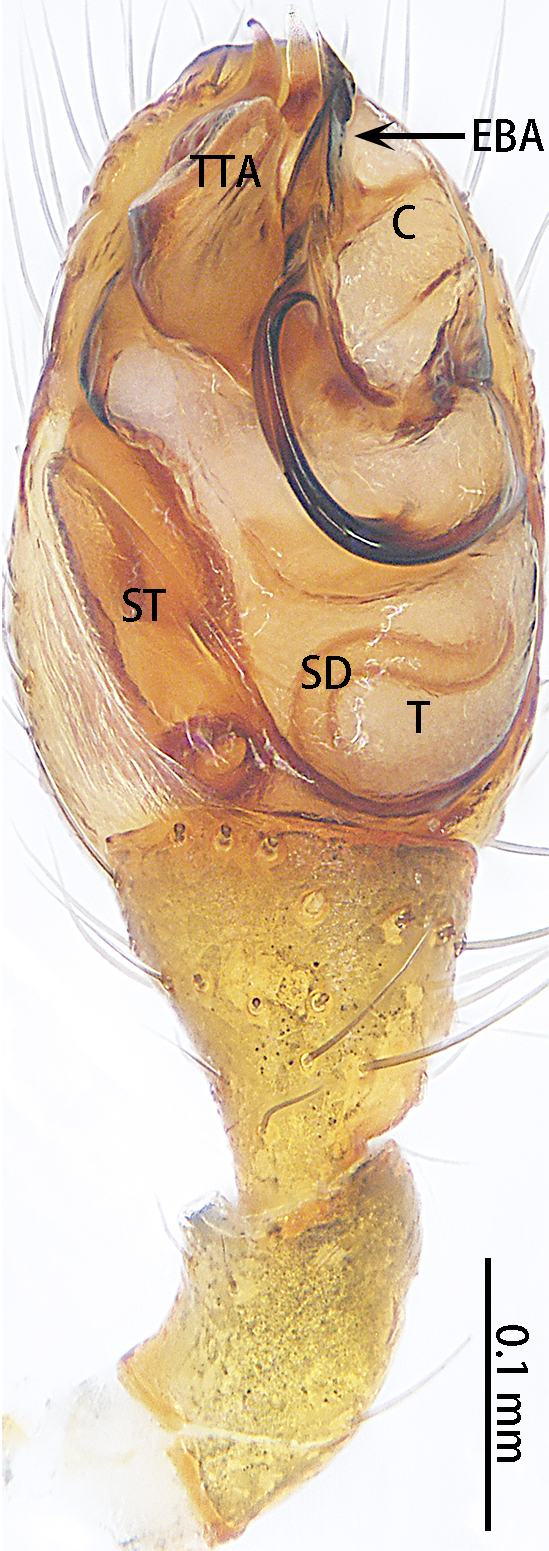
*S.nipponicus*.

**Figure 7a.**
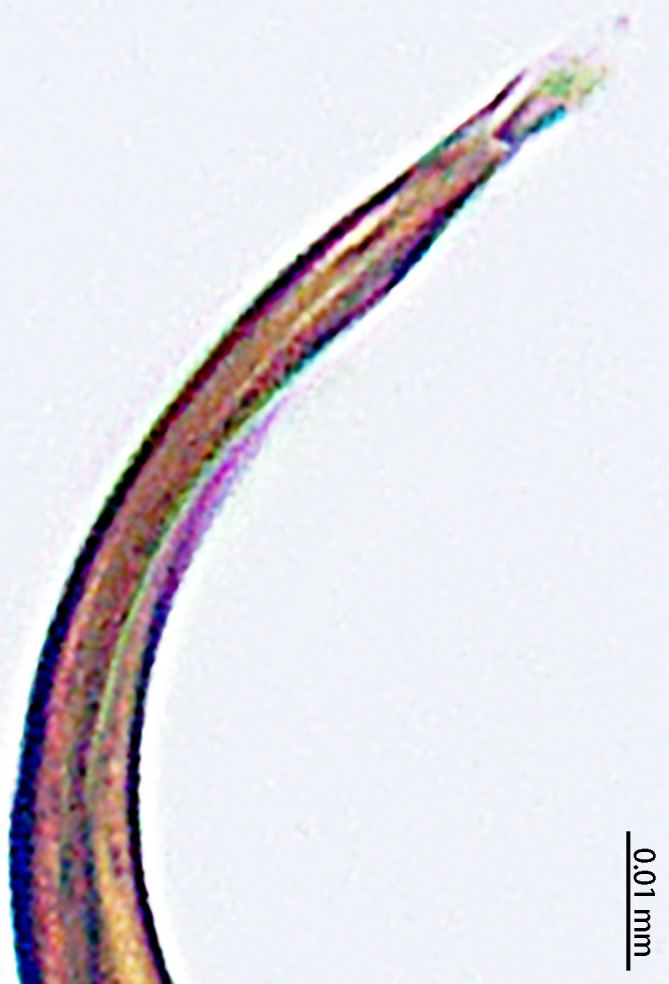
*S.forcipus*;

**Figure 7b.**
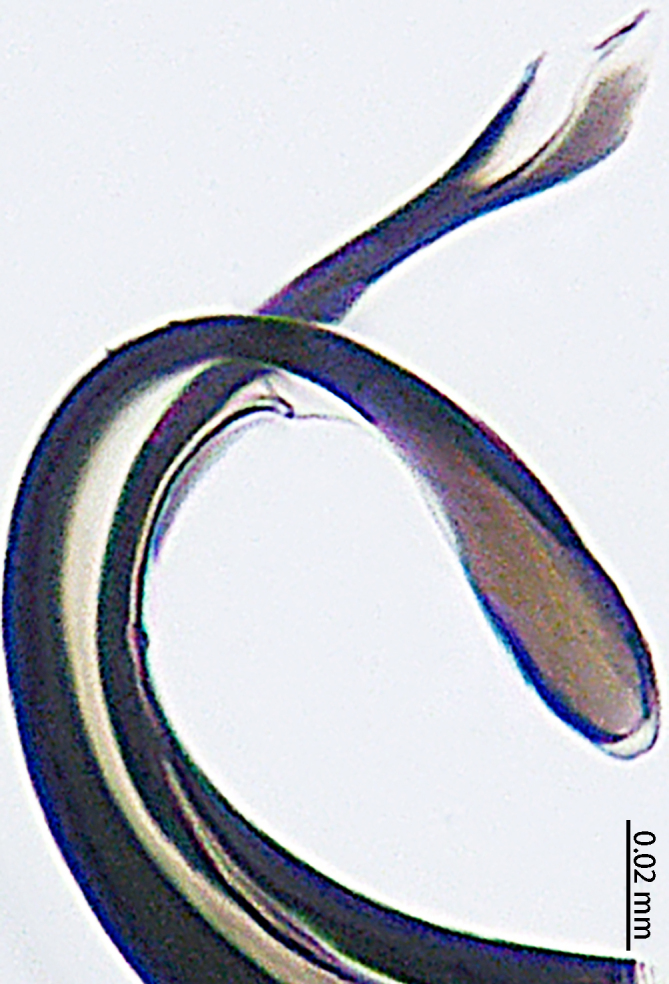
*S.lini* sp. n.;

**Figure 7c.**
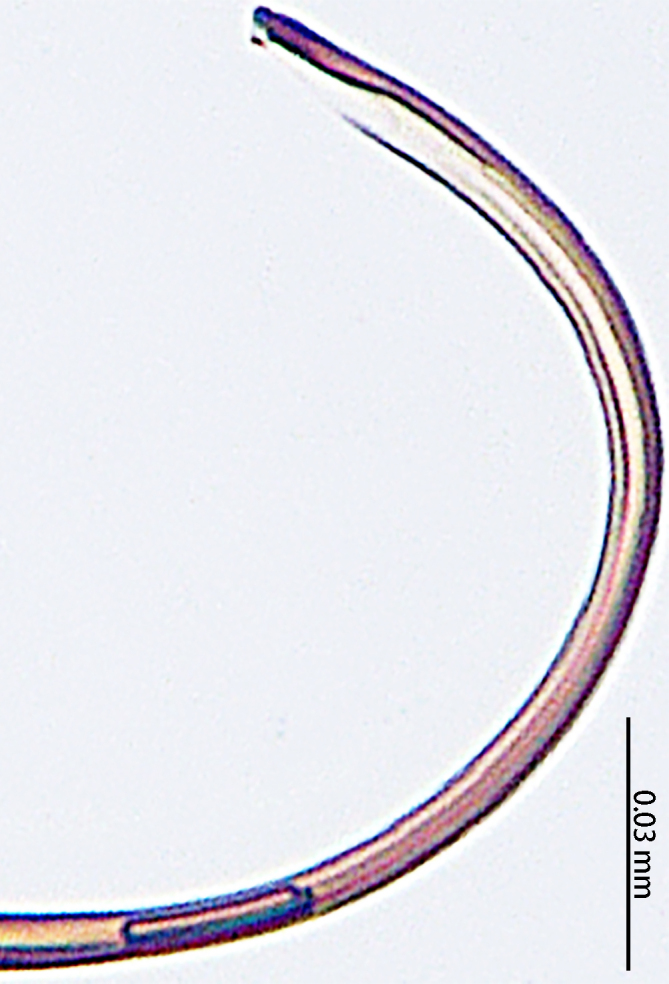
*S.nigrabdomenus*;

**Figure 7d.**
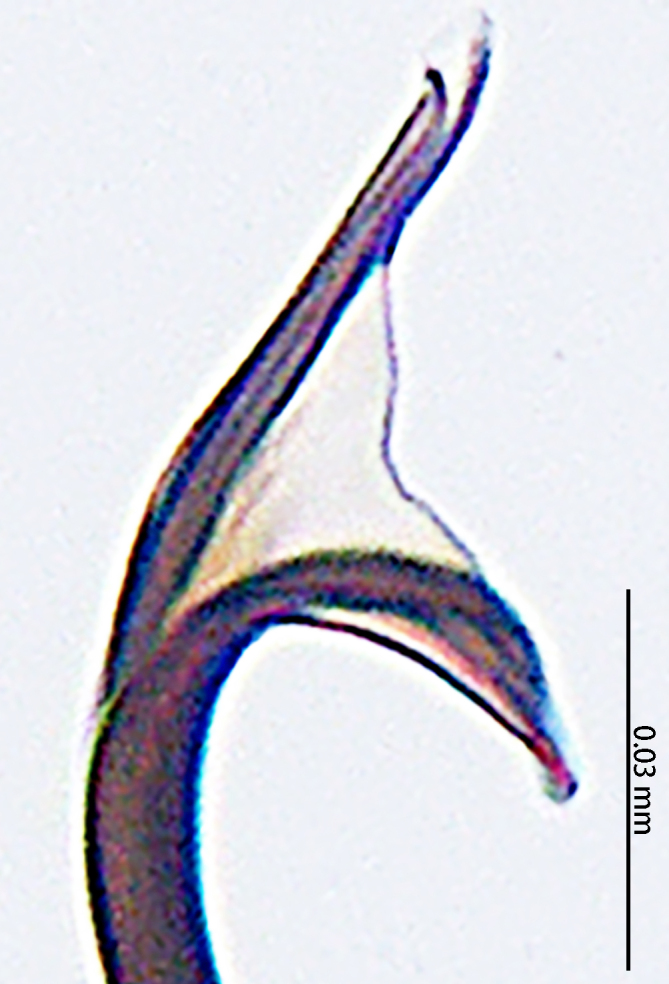
*S.nipponicus*.

**Figure 8a.**
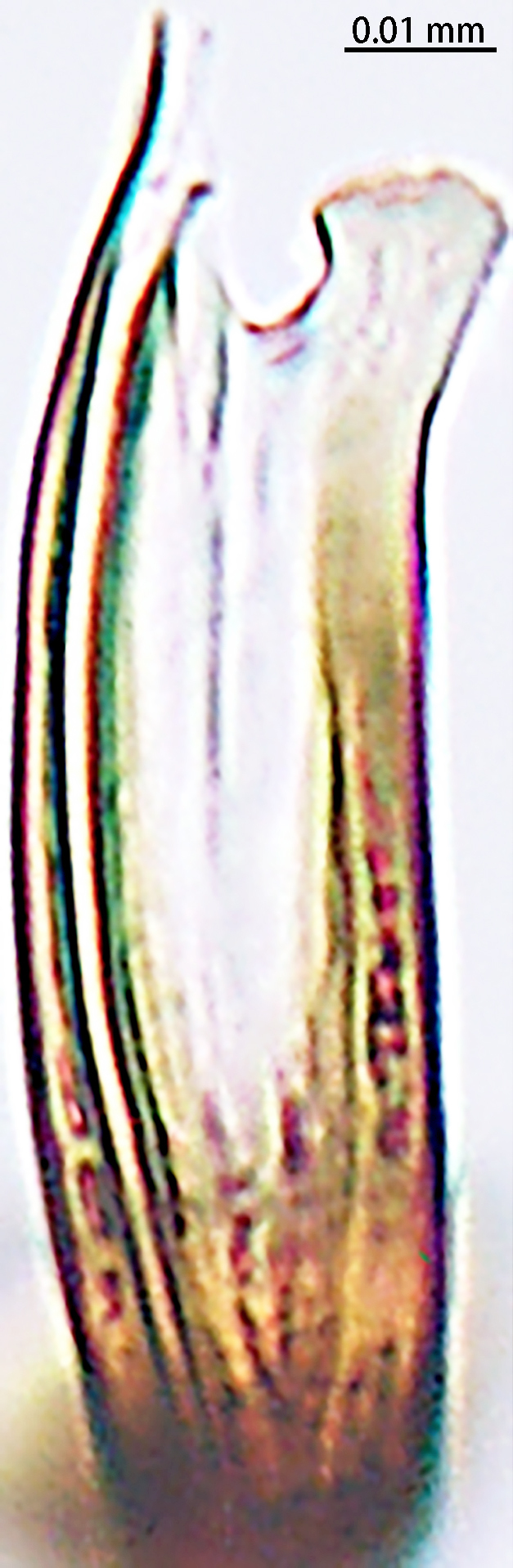
*S.forcipus*;

**Figure 8b.**
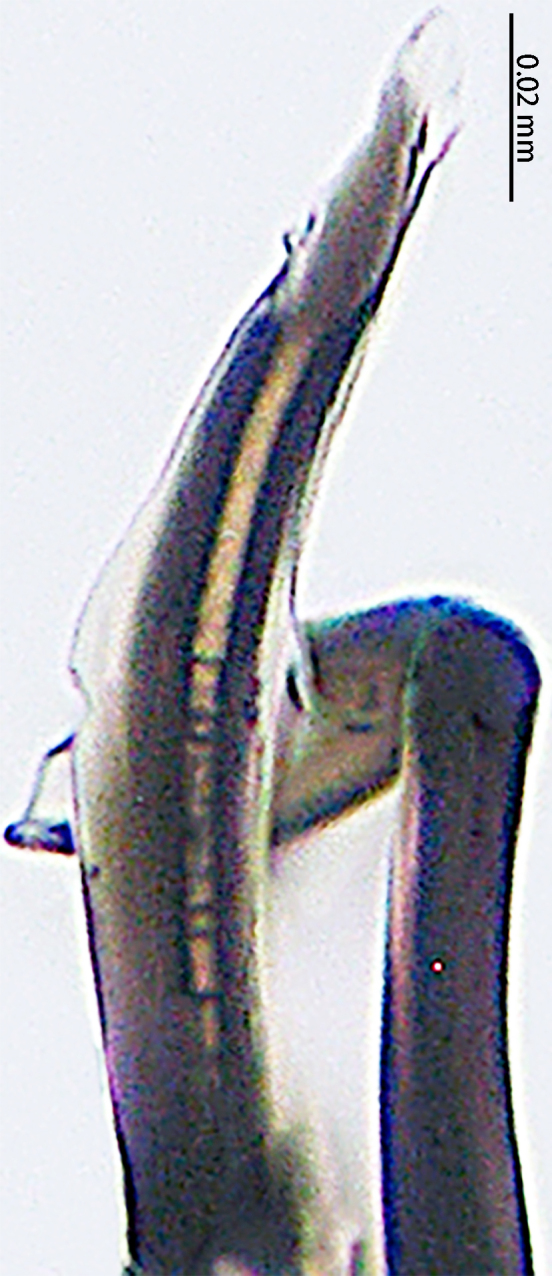
*S.lini* sp. n.;

**Figure 8c.**
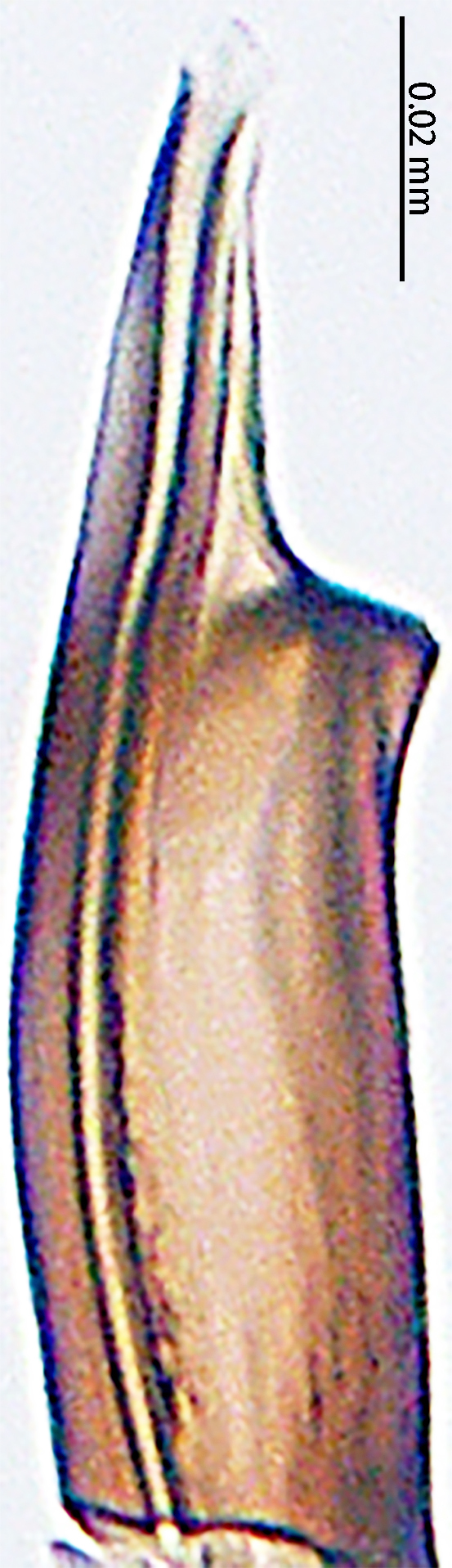
*S.nipponicus*.

**Figure 10a.**
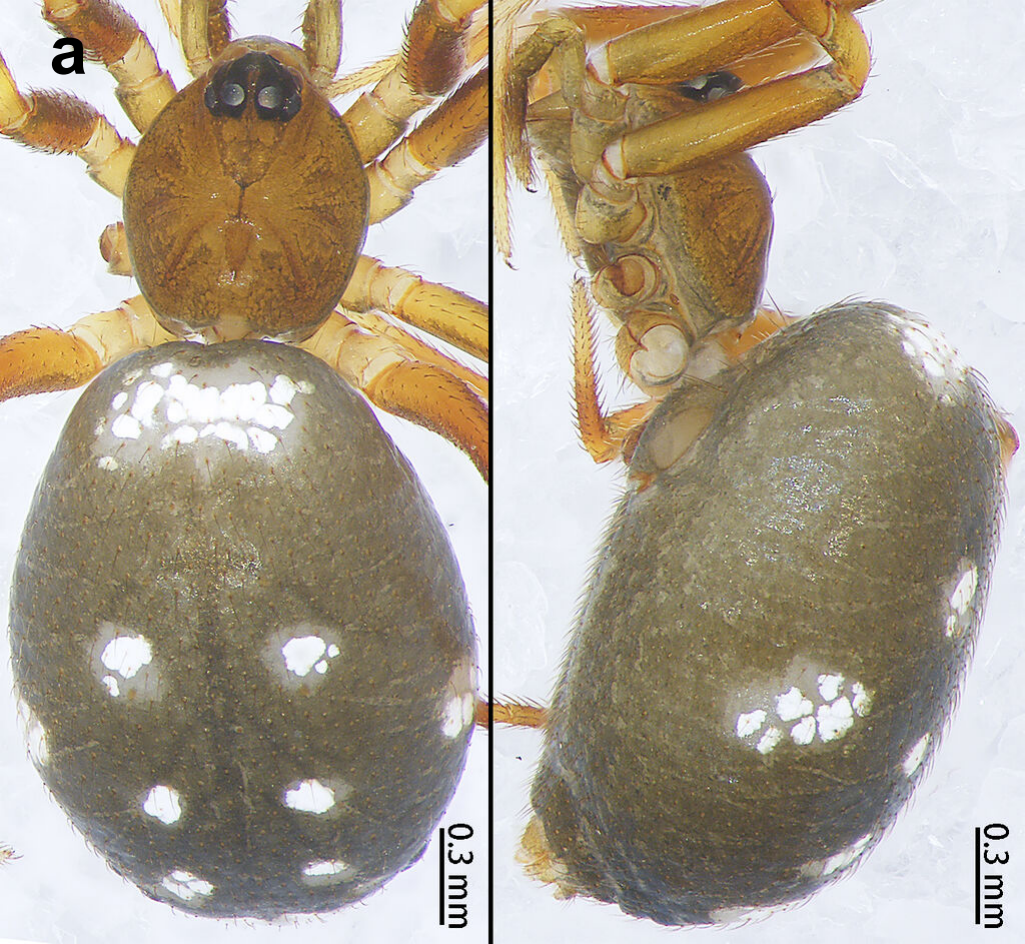
*S.atratus* sp. n., Female holotype. Dorsal (left photo); Lateral (right photo);

**Figure 10b.**
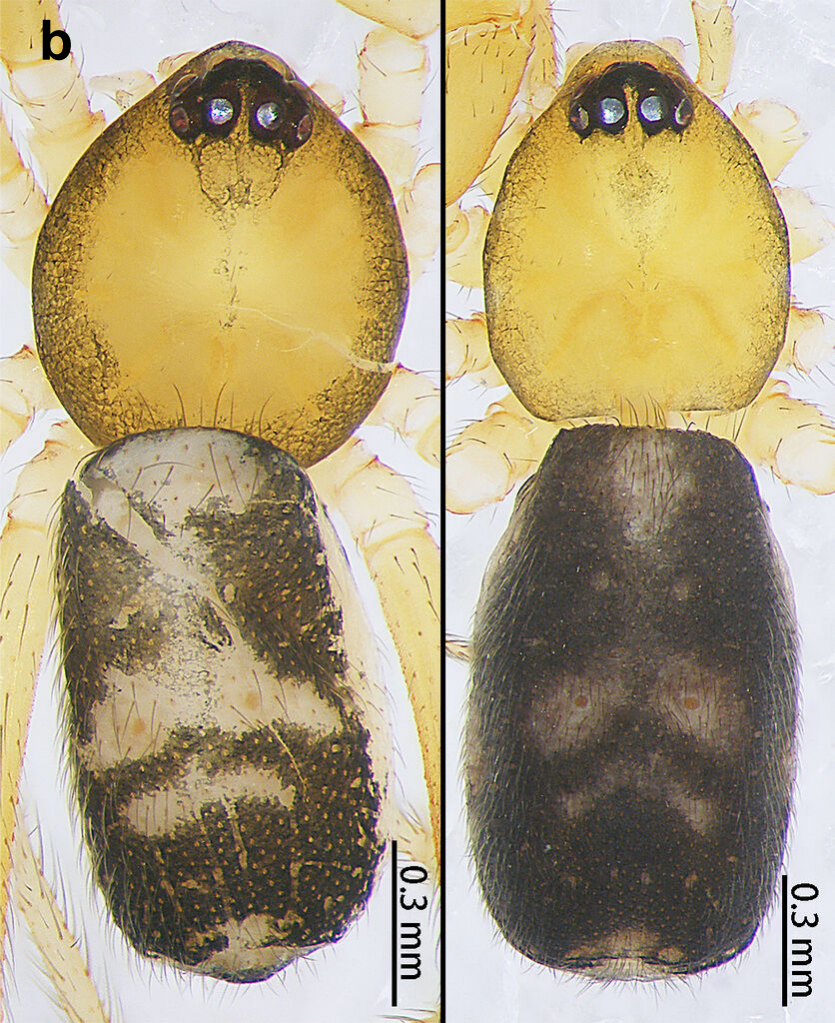
*S.lini* sp. n., dorsal. Male holotype (left photo); Female paratype (right photo);

**Figure 10c.**
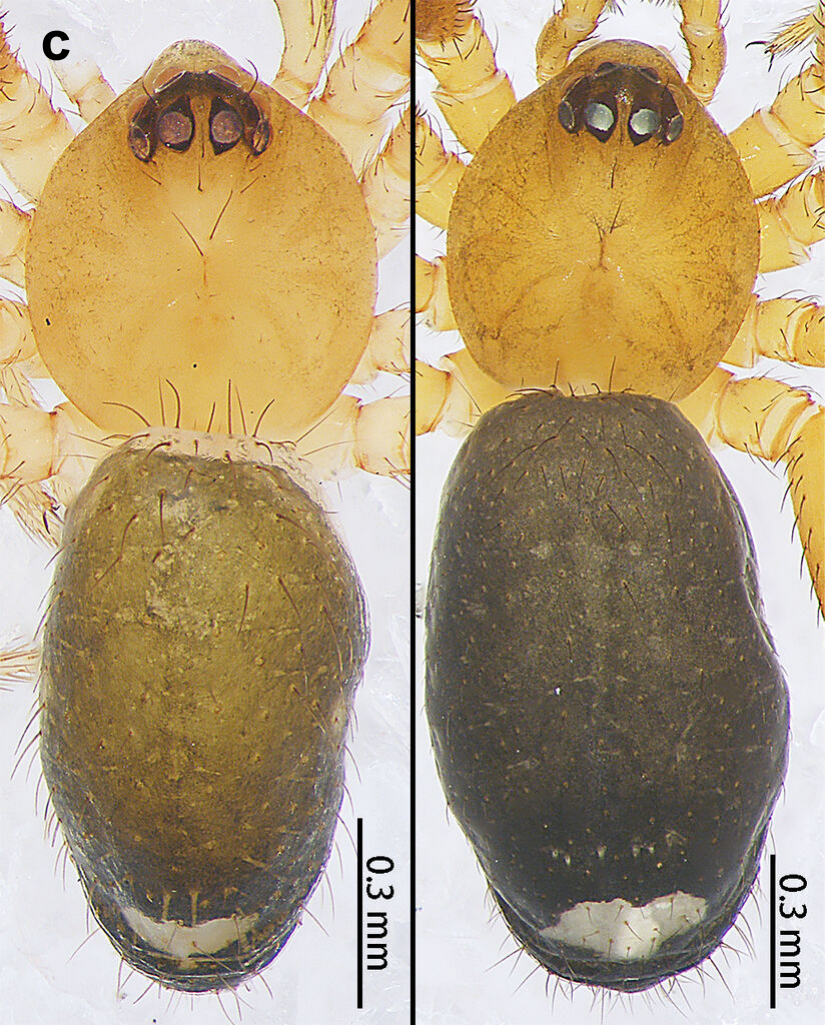
*S.nigrabdomenus*, dorsal. Male (left photo); Female (right photo).
